# A high plant density reduces the ability of maize to use soil nitrogen

**DOI:** 10.1371/journal.pone.0172717

**Published:** 2017-02-24

**Authors:** Peng Yan, Junxiao Pan, Wenjie Zhang, Junfang Shi, Xinping Chen, Zhenling Cui

**Affiliations:** 1 Key Laboratory of Plant-Soil Interactions, Ministry of Education, Center for Resources, Environment and Food Security, China Agricultural University, Beijing, China; 2 Key Laboratory of Tea Biology and Resources Utilization, Ministry of Agriculture, Tea Research Institute, Chinese Academy of Agricultural Sciences, Hangzhou China; Estacion Experimental del Zaidin, SPAIN

## Abstract

Understanding the physiological changes associated with high grain yield and high N use efficiency (NUE) is important when increasing the plant density and N rate to develop optimal agronomic management. We tested the hypothesis that high plant densities resulting in crowding stress reduce the ability of plants to use the N supply post-silking, thus decreasing the grain yield and NUE. In 2013 and 2014, a field experiment, with five N-application rates and three plant densities (6.0, 7.5, and 9.0 plants m^–2^), was conducted in the North China Plain (NCP). The calculated maximum grain yield and agronomic use efficiency (AE_N_) at a density of 7.5 plants m^–2^ were 12.4 Mg ha^–1^ and 39.3 kg kg^–1^, respectively, which were significantly higher than the values obtained at densities of 6.0 (11.3 Mg ha^–1^ and 30.2 kg kg^–1^) and 9.0 plant m^–2^ (11.7 Mg ha^–1^ and 27.8 kg kg^–1^). A high plant density of 9.0 plants m^–2^ decreased the post-silking N accumulation, leaf N concentration and net photosynthesis, which reduced the post-silking dry matter production, resulting in a low yield and NUE. Although a relatively low grain yield was observed at a density of 9.0 plants m^–2^, the optimal N rate increased from 150 to 186 kg N ha^-1^ at a density of 7.5 plants m^–2^. These results indicate that high plant densities with crowding stress reduce the ability of plants to use soil N during the post-silking period, and high rate of N fertilizer was needed to increase grain yield. We conclude that selecting the appropriate plant density combined with optimal N management could increase grain yields and the NUE in the NCP.

## Introduction

Studies conducted worldwide show that the maize yield potential per plant has remained unchanged for decades while the yield potential per unit area has increased as a function of higher population density and year of hybrid introduction [1–3]. These results often encourage producers to increase plant density to pursue high yields during maize production [4]. However, high plant densities, in practice, result in interplant competition that increases crowding stress, which has a detrimental effect on grain yield [5, 6]. In our former research with three plant densities (6.0, 7,5 and 9.0 plants m^–2^), the 9.0 plants m^–2^ reduced maize grain yield and NUE, which related to lower N uptake and biomass production and participation to grain during the grain-filling period [7]. Further understanding the changes in physiological traits with increasing plant density is necessary to improve our ability to achieve high productivity and resource efficiency.

Maize grain yield is determined by total biomass production [8], and some studies have documented that high biomass accumulation and nitrogen (N) uptake post-silking are the main contributors to high maize yields [9]. High plant densities result in interplant competition (particularly for light, water, and nutrients), which affects the vegetative and reproductive growth of maize [10]. Higher plant densities during the vegetative growth stage improve dry matter and N accumulation because of the large plant canopy [11]. However, a large plant canopy during the reproductive growth stage may accelerate the rate of leaf senescence, reducing post-silking dry matter accumulation and N uptake [11–14]. Therefore, the physiological changes associated with high grain yields and NUE that occur post-silking under conditions of high plant density must be better understood to improve agronomic management practices, including plant density and N fertilization.

Plant density and N fertilizer application are often considered the most important crop management practices to improve grain yield and N use efficiency (NUE) during intensive maize production [15, 16]. The yield and NUE decrease when maize is planted at a lower density because the plants grow to maturity without using all of the available soil N. However, plants compete for N at a higher density with crowding stress, thus decreasing the amount of N available per plant [6]. The crowding stress means high intraspecific competition for solar radiation, water, and soil nutrients [2]. When increases in plant density are insufficient to compensate for the reduced yield per plant (i.e., supra-optimal densities), crowding stress results in a lower yield and NUE [4, 13, 14, 17]. Lower plant N uptake and productivity per plant was observed in the high plant density, and more N fertilizer is essential for maximizing per plant N uptake and grain yield [13]. The critical question is whether adding more N can prevent this reduction in grain yield and NUE under conditions of high plant density.

In China, maize is produced under conditions of low plant density and high fertilizer input. In practice, a low plant density (5.0–6.0 plants m^–2^) and the over-application of N fertilizer (∼250 kg N ha^–1^), which restrict yields and NUE, are practiced by most farmers [18, 19]. In contrast, grain yields as high as 15.2 Mg ha^–1^ have been achieved in some studies under high plant densities (9.0–10.5 plants m^–2^) and N input (∼750 kg N ha^–1^) [20–22]. As a result, low NUE and substantial N losses were reported in these high-yielding studies [22].

We hypothesized that a high plant density with crowding stress would reduce the ability of plants to use available soil N. Integrated agronomic management, in terms of optimal plant density and N management, has the potential to improve maize grain yield and NUE. The objectives of this study were to: (1) evaluate grain yield and NUE at an optimal N rate under various plant densities; (2) compare biomass accumulation and N uptake pre- and post-silking at various plant densities and N rates; and (3) evaluate leaf area development and photosynthetic efficiency of leaf area post-silking.

## Materials and methods

### Field experiment

Field experiments were conducted in 2013 and 2014, in Quzhou (QZ) County (36.9°N, 115.0°E), which is part of Hebei Province in the North China Plain (NCP). No specific permission was required for these locations. This region has a warm, sub-humid, continental monsoon climate, with cold winters and hot summers. Annual cumulative mean temperature for days with mean temperatures above 10°C is 4000–5000°C, and the annual precipitation is 500–700 mm, with approximately 70% of the rainfall occurring during the maize growing season. Detailed weather information was described by Yan et al. (2016) [7]. In 2013 and 2014, the daily mean temperatures were 26 and 24°C, the total solar radiation was 1659 and 1807 MJ m^-2^ and the total precipitation was 351 and 274 mm, respectively. Winter wheat-summer maize is the primary crop rotation scheme used in this region. In 2013 and 2014, maize was planted without tillage after the winter wheat was harvested on 16 June and 11 June, and maize was harvested on 4 October and 3 October, respectively. No obvious weed or pest occurrences were observed during either growing season. The field studies did not involve endangered or protected species. The soil is clay loam, with a bulk density of 1.36 g cm^–3^, soil organic matter content of 14.2 g kg^–1^, total N content of 0.83 g kg^–1^, Olsen-phosphorus (Olsen-P) of 7.2 mg kg^–1^, and exchange potassium (K) of 125 mg kg^–1^ in the top 30 cm of the soil profile.

The experimental design was a split plot with four replicates. Five N treatments were applied as the main plot, and three plant densities were subplots. The size of the main plots was 300 m_2_ (15 m wide × 20 m long), and the subplot size was 100 m_2_ (5 m wide × 20 m long). The N treatments were as follows: control (0 N), 70% of optimal N rate (70%ONR), ONR based on in-season root-zone N management (INM, see below), 130% of ONR (130%ONR), and the typical N management practiced by farmers in the NCP (FNP). The FNP was set at 250 kg N ha^–1^ (100 kg N ha^–1^ applied at sowing and 150 kg N ha^–1^ applied at the six-leaf stage [V6]), which is typical for Hebei Province [18].

The ONR was determined as follows. The maize growth period was divided into three stages: sowing to V6, V6 to VT (silking stage), and VT to harvest. In 2013 and 2014, the V6 stage was on 19 July and 9 July, and the VT stage was on 10 August and 5 August, respectively. At sowing, 45 kg N ha^–1^ was applied. ONR was determined for the V6 to VT and the VT to harvest stages by subtracting measured soil nitrate-N content in the root zone (0–90 cm for the two growth stages) from the targeted N values. The targeted values for each growth period, which were estimated based on the targeted yield and maize N uptake, were 185 and 160 kg N ha^–1^, respectively. Urea was applied deep (10 cm) into the soil with a furrowing machine at the V6 and VT stages according to the N treatments, and rates of the different N treatments are listed in Table 1. Based on the soil P and K test results, all plots received appropriate amounts of triple superphosphate (P_2_O_5_ 45 kg ha^-1^) and potassium sulfate (K_2_O 90 kg ha^-1^) at sowing, accompanied by the application of urea.

**Table 1:** N fertilizer application rate (kg N ha^−1^) of different N treatments applied at sowing, six-leaf (V6) and silking (VT) stages during the two-year study. The N treatments included a no N control (0 N), the optimal N rate based on in-season root zone N management (ONR), 70% ONR, 130% ONR, and farmers’ standard N practice (FNP).

Treatment	2013			2014		
	At sowing	V6	VT	At sowing	V6	VT
0 N	0	0	0	0	0	0
70%ONR	32	21	62	32	62	29
ONR	45	30	88	45	88	41
130%ONR	59	39	114	59	114	53
FNP	100	150	0	100	150	0

A typical maize hybrid Zhengdan958 (ZD958), the most popular maize hybrid grown in China, was planted at three densities (6.0, 7.5, and 9.0 plants m^–2^), with a row spacing of 60 cm.

### Sampling and measurements

Five soil samples were taken in each plot at 30-cm increments to a depth of 90 cm before sowing, and 1 d before N application at the V6 and VT stages. Soil samples for the ONR treatments were extracted in a 1:1 ratio of soil to 0.01 mol L^–1^ CaCl_2_. Nitrate-N concentrations were measured using a nitrate-test strip and a reflectometer (Reflectometer RQflex, E. Merck, Darmstadt, Germany). These values were used to calculate ONR as the difference between the targeted N value and the measured soil nitrate-N content.

At the VT and R3 (milking stage) stage, the spike leaf photosynthesis (Pn) was measured with a portable photosynthesis apparatus (LI-6400, Li-Cor, Lincoln, NE, USA) in the late morning (09:00–11:00). The photon flux density, CO_2_ concentration, and temperature in the broadleaf chamber of the instrument (2 × 3 cm) were 1200 μmol m^–2^s^–1^, 380 μmol mol^–1^, and 25°C, respectively. Spike leaf samples from 10 plants were taken at the VT stage to measure leaf N concentration. At harvest, the aboveground biomass was estimated by clipping six plants in three rows near the middle of each plot, and the stover and kernels were separated. All samples were dried at 70°C in a forced-draft oven to a constant weight and subsequently weighed. Subsamples were passed through a 1-mm screen in a sample mill and mineralized using H_2_SO_4_-H_2_O_2_, after which the N concentration was measured using the Kjeldahl method [23]. At harvest, plants were removed manually from 5 × 2.4 m strips (four rows) in the middle of each plot.

### Data analysis

Maize grain yield response curves to N application rates for the various plant densities were generated using the NLIN procedure in the SAS software [24]. Three response models were evaluated: quadratic, quadratic-with-plateau, and linear-with-plateau [25]. The linear-with-plateau model produced the best fit. An analysis of variance (ANOVA) was performed using the SAS statistical analysis package (version 6.12, SAS Institute, Cary, NC, USA). A two-way ANOVA model, with N treatment (df = 4) and plant density (df = 2) as main effects, was used to assess the overall variability of biomass, grain and straw N concentration, N uptake, and N uptake efficiency (NupE). For the pre-and post-silking maize biomass accumulation and N uptake, and the spike leaf N concentration, differences were compared using the least-significant difference (LSD) test at the 0.05 probability level in SAS.

N uptake efficiency (NupE, kg kg^-1^), which represents the ability to take up N from the soil, was calculated as the aboveground N uptake (N_T_) divided by the amount of N fertilizer (N_F_). The agronomic use efficiency (AE_N_, kg kg^–1^) is the yield increase per unit of N applied. The N partial factor productivity (PFP_N_, kg kg^–1^) is the yield per unit of N fertilization. NupE, AE_N_, and PFPN) were calculated using Eqs (1)–(3).

$$\text{NupE}={\text{N}}_{\text{T}}/{\text{N}}_{\text{F}}$$ (1)

$${\text{AE}}_{\text{N}}=({\text{Y}}_{\text{N}}\text{-}{\text{Y}}_{0})/\text{N}$$ (2)

$${\text{PFP}}_{\text{N}}={\text{Y}}_{\text{N}}/\text{N}$$ (3)

where N_T_ and N_F_ are crop N uptake and rate of N application, Y_N_ and Y_0_ are grain yield with and without N application, and N is N rate.

## Results

### Grain yield and NUE at various N rates and plant densities

**Fig 1:**
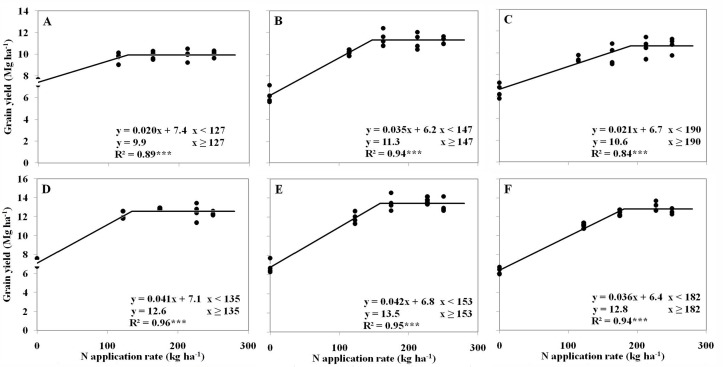
Maize grain yield as a function of N application rate for three plant densities during the two-year study. Planting densities were 6.0, 7.5, and 9.0 plant m^–2^ in 2013 in panels A–C, respectively, and were 6.0, 7.5, and 9.0 plant m^–2^ in 2014 panels D–F, respectively.

The responses of maize grain yield to increased N application rates were simulated via a linear-with-plateau model, at three densities and during two years (Fig 1; p < 0.001). As plant density increased from 6.0 to 7.5 plants m^–2^, the calculated average maximum grain yield in 2013–2014 increased from 11.3 to 12.4 Mg ha^–1^, and the calculated minimum N rate required to achieve the maximum grain yield increased from 131 to 150 kg N ha^–1^. With an increase in plant density to 9.0 plants m^–2^, the calculated minimum N rate required to achieve the maximum grain yield increased to 186 kg N ha^–1^; however, the maximum grain yield was only 11.7 Mg ha^–1^, which was 5.6% lower than that at 7.5 plants m^–2^.

Based on the stimulated linear-with-plateau model, the agronomic use efficiency (AE_N_) and the yield per unit of N fertilization (PFP_N_) were calculated at the inflection point (Fig 1). In 2013 and 2014, the AE_N_ averaged 39.3 kg kg^–1^ at 7.5 plants m^–2^, which was 30.0 and 40.9% higher than that for 6.0 (30.2 kg kg^–1^) and 9.0 (27.8 kg kg^–1^) plants m^–2^, respectively. The PFP_N_ values for densities of 6.0 and 7.5 plants m^–2^ reached a high overall ratio of 82.6–85.7 kg of grain per kilogram of N applied, which are notably higher than the 63.1 kg kg^–1^ for a density of 9.0 plants m^–2^.

The average ONR treatment based on in-season root-zone N management (INM) across both years was 169 kg N ha^–1^ (Table 1), which was 38 and 19 kg N ha^–1^ higher, and 19 N kg ha^–1^ lower than the calculated minimum N rates required to achieve the maximum grain yield for 6.0, 7.5, and 9.0 plants m^–2^, respectively. In both 2013 and 2014, the highest maize grain yields for densities of 6.0, 7.5, and 9.0 plants m^–2^ were achieved with the 70%ONR, ONR, and 130%ONR treatments, respectively.

### Dry matter and N accumulation

**Fig 2:**
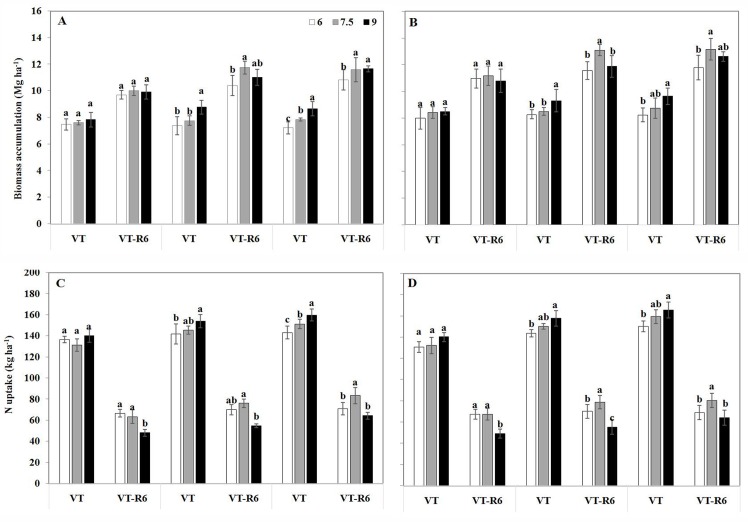
Pre-and post-silking maize biomass accumulation and N uptake (± S.E.) as affected by plant densities for various N-management schemes during the two-year study. Pre-and post-silking maize biomass accumulation in 2013 and 2014 in panels A–B, respectively, and pre-and post-silking maize N uptake in 2013 and 2014 in panels C–D, respectively. VT, silking stage; R6, harvest (maturity) stage, VT-R6 means the biomass accumulation and N uptake during the post-silking period. Lower case letters indicate significant differences between treatments at the P< 0.05 level.

Across all plant density and N treatments, biomass averaged 18.2 and 18.5 Mg ha^–1^ for 7.5 and 9.0 plants m^–2^, which were 5.5 and 7.2% higher than that for 6.0 plants m^–2^, respectively (Table 2). Although the average total biomass values were similar for densities of 7.5 and 9.0 plants m^–2^, the response of biomass accumulation pre- and post-silking to N rate differed between these two densities (Fig 2A and 2B). Before silking, the average biomass accumulation at 9.0 plants m^–2^ was 9.04 Mg ha^–1^, which was 11.3% higher than that for 7.5 plants m^–2^. After silking, the biomass accumulation post-silking at a density of 7.5 plants m^–2^ was 12.4 Mg ha^–1^ for the ONR treatment, which was 7.8% higher than the biomass accumulation at 9.0 plants m^–2^. Across all five N treatments, the highest biomass values for densities of 6.0, 7.5, and 9.0 plants m^–2^ were 18.9, 20.6, and 21.4 Mg ha^–1^, respectively, which were achieved with the 70%ONR, ONR, and 130%ONR treatments, respectively.

**Table 2:** Maize biomass, grain and straw N concentration, N uptake, and uptake efficiency at harvest in 2013 and 2014 for plant densities of 6.0, 7.5, and 9.0 plants m^–2^, and all N treatments. N treatments included a no N control (0 N), optimal N rate based on in-season root zone N management (ONR), 70%ONR, 130%ONR, and farmers’ standard N practice (FNP).

Year	Treatment	Biomass (Mg ha^–1^)			Grain N (%)			Straw N (%)			N uptake (kg N ha^–1^)			N uptake efficiency N		
		6	7.5	9	6	7.5	9	6	7.5	9	6	7.5	9	6	7.5	9
2013	0 N	11.1	10.9	11.8	0.95	0.92	0.89	0.55	0.59	0.48	81	80	77			
	70%ONR	17.2	17.6	17.9	1.20	1.23	1.20	1.03	0.88	0.81	183	190	174	1.60	1.61	1.54
	ONR	17.8	19.5	19.8	1.33	1.33	1.30	1.05	1.06	0.99	200	229	214	1.22	1.40	1.31
	130%ONR	18.0	19.4	20.0	1.44	1.35	1.30	1.05	1.17	0.97	211	234	224	1.00	1.10	1.05
	FNP	19.2	18.9	20.1	1.44	1.33	1.27	1.06	1.05	1.06	207	222	222	0.83	0.89	0.89
N		***			***			***			***			***		
Density		***			**			**			**			**		
N × Density		ns			ns			ns			ns			ns		
2014	0 N	10.5	11.0	11.3	0.85	0.76	0.71	0.48	0.49	0.38	82	84	87			
	70%ONR	18.9	19.6	19.3	1.07	1.02	0.96	0.84	0.76	0.64	194	199	189	1.60	1.64	1.56
	ONR	19.8	21.6	21.2	1.18	1.09	1.04	0.91	0.85	0.78	211	229	213	1.22	1.32	1.23
	130%ONR	20.0	21.9	22.3	1.28	1.11	1.04	0.91	0.97	0.77	215	240	230	0.96	1.07	1.02
	FNP	20.1	21.7	21.3	1.28	1.10	1.02	0.92	0.87	0.84	217	229	226	0.87	0.92	0.90
N		***			***			***			***			***		
Density		***			***			**			**			**		
N × Density		ns			ns			ns			ns			ns		

** *P*<0.01

*** *P*<0.001

ns not significant

Across all N treatments and two years, the highest N uptake of 194 kg N ha^–1^ was observed for 7.5 plants m^–2^ in contrast with 180 and 186 kg N ha^–1^ for densities of 6.0 and 9.0 plants m^–2^, respectively (Table 2). Lower grain and straw N concentration values were observed for 9.0 plants m^–2^ than that for 6.0 or 7.5 plants m^–2^. The grain N concentration was 1.07 g kg^–1^ at a density of 9.0 plants m^–2^, which was 4.5 and 10.8% lower than that for densities of 7.5 and 6.0 plants m^–2^, respectively. The straw N concentrations at 6.0 and 7.5 plants m^–2^ were 0.88 and 0.87 g kg^–1^, which were14.3 and 13.0%, respectively, higher than that at 9.0 plants m^–2^ (0.77 g kg^–1^)^.^

Variation in N uptake pre- and post-silking was observed for the N treatments and plant densities (Fig 2C and 2D). For the ONR treatment, we observed 5.4% higher N uptake pre-silking at a density of 9.0 plants m^–2^ than that at 7.5 plants m^–2^ (148 kg N ha^–1^). However, the N uptake post-silking at 9.0 plants m^–2^ was 55 kg N ha^–1^, which was 28.6% less than the uptake of 77 kg N ha^–1^ at 7.5 plants m^–2^. Correspondingly, the highest N uptake efficiency was achieved at 7.5 plants m^–2^, which was 7.0 and 4.7% higher than the values of 1.16 and 1.19 achieved at 6.0 and 9.0 plants m^–2^, respectively.

### Leaf area development and photosynthetic efficiency of the spike leaf at post-silking

Both the maize leaf area index (LAI) and spike leaf photosynthesis (Pn) at the silking (VT) and milking (R3) stages were affected significantly by plant density (Table 3). Across the two growing seasons, the average LAI values at 7.5 plants m^–2^ during the VT and R3 stages were 5.01 and 4.79, respectively, which were 21.9 and 22.8% higher, respectively, than that at 6.0 plants m^–2^. At a plant density of 9.0 plants m^–2^, the LAI values at the VT and R3 stages exhibited additional increases of only 9.6 and 7.5%, respectively, relative to 7.5 plants m^–2^. There were no significant differences in Pn between the densities of 6.0 and 7.5 plants m^–2^, which were 36.0 and 34.6 μmol m^–2^ s^–1^ at the VT stage, and 26.1 and 25.3 μmol m^–2^ s^–1^ at the R3 stage, respectively. Increasing the plant density from 7.5 plants m^–2^ to 9.0 plants m^–2^ reduced the average Pn by 5.5% from 34.6 to 32.8 μmol m^–2^ s^–1^ and by 8.2% from 25.3 to 23.4 μmol m^–2^ s^–1^ at the VT and R3 stages, respectively.

**Table 3:** Maize leaf area index (LAI) and spike leaf photosynthesis (Pn) as affected by three plant densities under the 70%ONR, ONR, and 130%ONR N management schemes at the silking (VT) and milking stages (R3) during the two-year study.

		LAI at VT			Pn at VT			LAI at R3			Pn at R3		
		6	7.5	9	6	7.5	9	6	7.5	9	6	7.5	9
2013	70%ONR	3.74	4.52	4.73	40.5	36.9	34.5	3.48	4.40	4.54	26.7	23.5	22.0
	ONR	3.79	4.71	5.13	41.5	40.2	38.5	3.64	4.59	4.99	30.2	29.2	27.1
	130%ONR	3.89	4.70	5.16	41.0	40.1	39.5	3.74	4.66	5.08	30.3	29.5	28.8
	N	*			**			*			**		
	Density	***			**			***			***		
	N× Density	ns			*			ns			*		
2014	70%ONR	4.05	4.81	5.36	29.2	26.6	25.0	3.86	4.38	4.72	21.7	20.2	18.6
	ONR	4.43	5.31	5.84	30.4	29.1	27.0	4.16	4.98	5.32	22.0	21.4	19.8
	130%ONR	4.35	5.32	5.90	31.7	30.6	28.7	4.23	4.99	5.40	23.4	22.1	21.1
	N	*			**			*			**		
	Density	***			***			***			***		
	N× Density	ns			*			ns			*		

* *P*<0.05

** *P*<0.01

*** *P*<0.001

ns not significant

We observed different responses of maize LAI and Pn to N treatment among the plant densities (Table 3). At a density of 6.0 plants m^–2^, in both 2013 and 2014, the highest LAI and Pn values at the VT and R3 stages were achieved with the 70%ONR treatment, except for the highest Pn, which was achieved with ONR treatment at the R3 stage in 2013. As the plant density increased from 6.0 to 7.5 plants m^–2^, the N application rate needed to increase to the ONR treatment to achieve the highest LAI and Pn values at the VT and R3 stages in 2013 and 2014. With the plant density increased to 9.0 plants m^–2^, the highest LAI values at the VT and R3 stages were achieved with the ONR treatment. However, the N application rate of the 130%ONR treatment was needed to achieve the highest Pn at the VT and R3 stages.

**Fig 3:**
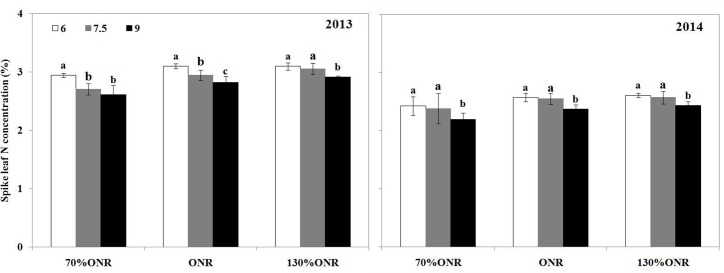
Maize spike leaf N concentration as affected by three plant densities under the 70%ONR, ONR, and 130%ONR N-management schemes at the silking stage during the two-year study. Lower case letters indicate significant differences between treatments at the P< 0.05 level.

The spike leaf N concentration at the VT stage was affected significantly by plant density and N management in both 2013 and 2014 (Fig 3). Across the two growing seasons, the spike leaf N concentration decreased from 2.80 g kg^–1^ at 6.0 plants m^–2^ to 2.70 and 2.55 g kg^–1^ at 7.5 and 9.0 plants m^–2^, respectively (Fig 3). The highest average spike leaf N concentration values at the VT stage for densities of 6.0, 7.5, and 9.0 plants m^–2^ were 2.82, 2.75, and 2.69 g kg^–1^ for the 70%ONR, ONR, and 130%ONR treatments, respectively.

## Discussion

Adjusting plant density has been an important agronomic practice for enhancing the yield and NUE of maize worldwide [4]. In this study, the highest maize grain yield and AE_N_ values of 12.4 Mg ha^–1^ and 39.3 kg kg^-1^, respectively, were achieved at a density of 7.5 plants m^–2^, which were notably higher than the values at densities of 6.0 and 9.0 plants m^–2^, respectively (Fig 1). Normally, the 6.0 plants m^–2^ is the normal plant density adopted by farmers in NCP, the 7.5 plants m^–2^ is the recommended plant density by the agricultural science and technological workers. Compared with the average grain yield and AE_N_ values of 7.3 Mg ha^–1^ and 11.0 kg kg^–1^, respectively, for the NCP, the corresponding values for 7.5 plants m^–2^ were 69.9 and 266.4% higher [26, 27].

Maize grain yield is largely influenced by the balance of assimilate allocation between vegetative and reproductive organs [11]. Our former research found the total biomass accumulation were similar between the optimal and supra-optimal plant densities, however, lower harvest index (HI) was observed with the supra-optimal plant density, which lead to lower grain yield [7]. Some studies have found a positive relationship between maize grain yield and post-silking N accumulation [28–30], but other studies have found no relationship between post-silking N accumulation and yield [31]. Based on 86 published field experiments, Mueller and Vyn reported that post-silking N accumulation is more strongly correlated with grain yield when plants are under severe N stress than under less acute N stress at silking [32].

In the present study, although the total N uptake rates were similar, high plant densities reduced post-silking N accumulation by 40% at densities over 7.5 plants m^–2^ (Fig 2B). Post-silking dry matter accumulation at a density of 9.0 plants m^–2^ decreased by 8% compared to a density of 7.5 plants m^–2^. Remobilization of assimilates stored in vegetative tissues is usually very low in maize [12, 33]. The reduced post-silking dry matter and N accumulation at a density of 9.0 plants m^–2^ reduced the ability of the plants to use the available supply of soil N (Table 2); this decreased the grain yield and NUE. Yan et al reported relative to low-yield maize, high-yield maize had higher biomass and N accumulation, especially during the grain-filling period [7]. Therefore, improving the N uptake and biomass accumulation during the post-silking period through breeding or N management is vital to improve maize grain yield and NUE.

Previous studies suggested that the increase in total N uptake is largely driven by increased biomass accumulation [34]. Other studies indicated that N and dry matter accumulation during the grain-filling period are not as tightly linked [32]. Our results indicate that carbon assimilate availability post-silking is likely not the sole, critical variable determining yield differences between optimal and high plant densities, whereas the leaf N concentration and quantity of N may have an important role in yield. An increase in the quantity of N at this stage allows high net photosynthesis to be sustained for a longer period to increase the conversion of solar radiation and carbon accumulation.

In the present study, as the plant density increased from 6.0 to 7.5 plants m^–2^, the average LAI at the VT and R3 stages increased and no significant reductions in Pn were observed (Table 3). At a plant density of 9.0 plants m^–2^, the leaf N concentration and Pn decreased, reducing post-silking production and resulting in a lower grain yield. Similar results suggested that excess plant density often increases inter-plant competition, causes leaf shading, and decreases the amount of resources available per plant [6], thereby reducing canopy photosynthesis [35], accelerating leaf senescence, and reducing the grain yield and NUE [36].

Although the maximum grain yield was low at a density of 9.0 plants m^–2^ compared to that at 7.5 plants m^–2^, the corresponding N requirement rate increased from 150 to 186 kg N ha^-1^. This result further supports the notion that a high density reduces the ability of plants to use available soil N, which related to lower N uptake efficiency (Fig 2), and lower spike leaf N concentration (Fig 3) at post-silking period. This result also partly explains why a high N rate was applied at a high plant density in some high-yielding studies in China (∼750 kg N ha^–1^; 9.0–10.5 plants m^–2^) [22].

Previous studies have reported that lower plant density and excessive use of N fertilizer are the main contributors to lower grain yield and NUE in the NCP [18, 27]. According to our research, increasing the plant density from 6.0 to 7.5 plants m^–2^ improved both grain yield and NUE. However, further increases to 9.0 plants m^–2^ caused grain yield and NUE reductions. In this research, this implies that the 7.5 plants m^–2^ is the optimal plant density to achieve both high grain yield and NUE without variety alternative in NCP. In practice, the optimal plant density for high grain yield and NUE might be affected by maize cultivars, climate conditions, N management practices, and perhaps other factors [11, 14, 37–39]. Normally, compared to old maize cultivars, new maize cultivars are more tolerance to crowding stress and higher plant density is necessary to achieve its highest grain yield, and increased rate of N application is necessary to meet the demand of N [11]. What is more, the optimal plant density to achieve its highest grain yield was determined by the climate condition [22]. In our research, the 7.5 plants m^–2^ is similar to the optimal plant density in America [11], while it is lower than that 9.0–10.5 plant m^-2^ for the high yield study in China [37–39]. Future additional research that compares maize cultivars, plant densities, N management practices, and climate conditions would be useful to guide maize production in the NCP.

## Conclusion

Increased both plant densities and the use of N fertilizer in the last 10 years have been considered the main practical strategies to improve maize grain yield for Chinese maize production. In this study, the highest maize grain yield and N partial factor productivity (PFP_N_) were 12.4 Mg ha^–1^ and 82.6 kg kg^–1^, respectively, which were achieved at 7.5 plants m^–2^ under the optimal N rate (ONR) treatment. Further increase the plant densities to 9.0 plants m^–2^ reduced the ability of plants to use the available soil N supply post-silking, thus decreasing the biomass and N accumulation post-silking with low leaf N concentrations and net photosynthesis, resulting in a low grain yield and N use efficiency (NUE). Low plant densities (6.0 plants m^–2^) decreased the biomass and N accumulation due to a low leaf area index, resulting in a low grain yield and NUE. Among the five N treatments tested, the highest maize grain yields for densities of 6.0, 7.5, and 9.0 plants m^–2^ were achieved using a 70%ONR treatment, an ONR treatment based on in-season root-zone N management, and a 130%ONR treatment, respectively. According to the characteristics of maize cultivars and soil N supply, choosing the the 7.5 plants m^–2^ plant density and the optimal N management based on in-season root-zone N management could contribute to higher grain yield and NUE in the North China Plain.
